# Emergence and Spread of *Streptococcus pneumoniae* with *erm*(B) and *mef*(A) Resistance

**DOI:** 10.3201/eid1106.050222

**Published:** 2005-06

**Authors:** David J. Farrell, Stephen G. Jenkins, Steven D. Brown, Manish Patel, Bruce S. Lavin, Keith P. Klugman

**Affiliations:** * G.R. Micro Ltd, London, United Kingdom;; † Mount Sinai School of Medicine, New York, New York, USA;; ‡ Clinical Microbiology Institute, Wilsonville, Oregon, USA;; § sanofi-aventis, Bridgewater, New Jersey, USA;; ¶ Emory University, Atlanta, Georgia, USA;; # University of the Witwatersrand, Johannesburg, South Africa

**Keywords:** human papillomavirus, Cervix neoplasms, Cost-benefit analysis, vaccines, public health

## Abstract

*Streptococcus pneumoniae* isolates (N = 31,001) were collected from patients with community-acquired respiratory tract infections during the PROTEKT US surveillance study (2000–2003). While the macrolide (erythromycin) resistance rate remained stable at ≈29%, the prevalence of resistant isolates containing both *erm*(B) and *mef*(A) increased from 9.7% in year 1 to 16.4% in year 3, with substantial regional variability. Almost all (99.2%) dual *erm*(B)+*mef*(A) macrolide-resistant isolates exhibited multidrug resistance, whereas 98.6% and 99.0% were levofloxacin- and telithromycin-susceptible, respectively. These strains were most commonly isolated from the ear or middle-ear fluid of children. Of 152 representative *erm*(B)+*mef*(A) isolates, >90% were clonally related to the multidrug-resistant international Taiwan^19F^-14 clonal complex 271 (CC271). Of 366 *erm*(B)+*mef*(A) isolates from the PROTEKT global study (1999–2003), 83.3% were CC271, with the highest prevalence seen in South Africa, South Korea, and the United States. This study confirms the increasing global emergence and rapidly increasing US prevalence of this multidrug-resistant pneumococcal clone.

*Streptococcus pneumoniae* is a key pathogen implicated in community-acquired respiratory tract infections, including acute otitis media (1), community-acquired pneumonia (2), acute exacerbations of chronic bronchitis (3), and acute bacterial sinusitis (4). During the last decade, the clinical management of respiratory infections has become increasingly complicated by the emergence and spread of resistance in *S. pneumoniae* to commonly used antibacterial drugs, particularly β-lactams and macrolides, both in the United States (5–10) and worldwide (11–13). PROTEKT (Prospective Resistant Organism Tracking and Epidemiology for the Ketolide Telithromycin) is an international, longitudinal surveillance study initiated in 1999 to evaluate the activity of telithromycin, a new ketolide antibacterial drug, against *S. pneumoniae* and other common respiratory pathogens and to compare its activity with other antibacterial drugs (13). In addition, the integration of genotypic testing into PROTEKT has helped elucidate the international molecular epidemiology of resistant strains (14,15).

PROTEKT US is a sister program to the PROTEKT global study that was initiated in 2000, specifically to monitor antibacterial resistance in the United States. Data from PROTEKT US showed an overall pneumococcal macrolide (erythromycin) resistance rate of 31.0% in 2000 and 2001 (9). Macrolide resistance in *S. pneumoniae* is mediated by 2 major mechanisms: methylation of ribosomal macrolide target sites, encoded by the *erm*(B) gene, and drug efflux, encoded by *mef*(A) (14–17). While *erm*(B) is the dominant genotype across much of the world, *mef*(A)-mediated mechanisms of resistance predominate in the United States (14). Recently, PROTEKT and other studies have identified *S. pneumoniae* isolates with both *erm*(B) and *mef*(A) genes in the United States, Canada, South Korea, China, South Africa, Japan, Mexico, and Hungary (14,15,18–22). The initial confirmation of isolates with both *erm*(B) and *mef*(A) was first described in the South African study (19). These dual *erm*(B)+*mef*(A) isolates belong predominantly to 1 major clonal complex (15) and show high rates of resistance to multiple classes of antibacterial drugs; consequently, their potential spread is of serious concern.

We report the prevalence of the multidrug-resistant *erm*(B)+*mef*(A) clonal complex in the United States. In addition, molecular epidemiologic data for macrolide-resistant *S. pneumoniae* isolates collected as part of the PROTEKT US study from 2000 to 2003 are compared with data for isolates collected as part of the PROTEKT global study (1999–2003) to assess the spread of the *erm*(B)+*mef*(A) clonal complex.

## Methods

For the PROTEKT US study, isolates of *S. pneumoniae* were collected from across the United States from 2000 to 2003. The numbers of collection centers that provided samples were 207 in year 1 (2000–2001), 241 in year 2 (2001–2002), and 247 in year 3 (2002–2003).

Pathogenic respiratory tract isolates of *S. pneumoniae* were collected from adult and pediatric outpatients with community-acquired respiratory tract infections (acute otitis media, pneumonia, acute exacerbations of chronic bronchitis, acute exacerbations of chronic obstructive pulmonary disease, and sinusitis). Also included were isolates cultured from material collected from hospitalized patients within 48 hours of admission. The following sources were considered acceptable: cultures from blood, sputum, bronchoalveolar lavage, middle-ear fluid (collected by tympanocentesis), nasopharyngeal swab or aspirate, and sinus aspirate. Patients with nosocomial respiratory tract infections and those with cystic fibrosis were excluded. Duplicate strains, or strains originating from existing collections, were also not included in the study. Demographic data collected included the age and sex of the patient, infection, culture source, inpatient versus outpatient status, specimen accession number, and date of sample collection. Details of the methods for isolate storage, transportation, and identification have been reported previously (23).

MICs were determined at a central laboratory (CMI, Portland, OR, USA) by using the Clinical and Laboratory Standards Institute (CLSI) broth microdilution method (24). The following antibacterial agents were tested: amoxicillin-clavulanate (amoxicillin alone was not tested; however, susceptibility can be extrapolated from the amoxicillin-clavulanate results), azithromycin, cefuroxime, clarithromycin, clindamycin, co-trimoxazole, erythromycin, levofloxacin, linezolid, penicillin, telithromycin, and tetracycline. In all cases, CLSI MIC interpretive criteria were used to define susceptibility and resistance (25). Susceptibility to telithromycin was determined by using the CLSI breakpoints (25): susceptible ≤1 μg/mL; intermediate 2 μg/mL; resistant ≥4 μg/mL.

All erythromycin-resistant (MIC ≥1 μg/mL) pneumococcal isolates collected from PROTEKT US years 1–3 were analyzed for the presence of *erm*(B), *erm*(A) subclass *erm*(TR), and *mef*(A) macrolide resistance genes. Isolates in year 1 were analyzed by multiplex rapid-cycle polymerase chain reaction (PCR) with microwell-format probe hybridization, as described previously (26). In years 2 and 3, isolates were analyzed by using a multiplex TaqMan (Applied Biosystems, Foster City, CA, USA) PCR assay that was validated against the previous PCR method (27).

A proportion of dual *erm*(B)+*mef*(A) macrolide-resistant isolates underwent serotyping and multilocus sequence type (MLST) determination at G.R. Micro Ltd (London, UK). Isolates were serotyped by using antisera from the Statens Serum Institute (SSI, Copenhagen, Denmark). MLST was determined as described previously (15).

Serotyping and MLST determination were also conducted on 366/378 dual *erm*(B)+*mef*(A) macrolide-resistant *S. pneumoniae* isolates, respectively, collected from the global PROTEKT study (1999–2003). Sequence type (ST) and alleles were analyzed by UPGMA (unweighted pair group method with arithmetic mean) and BURST (based upon related STs) analysis by using the START program (version 1.0.5 [28]) to assign lineage and clonal complexes.

## Results

### Macrolide Resistance Mechanisms

From 2000 to 2003, a total of 31,001 *S. pneumoniae* isolates were collected as part of the PROTEKT US study: 10,103 in year 1, 10,012 in year 2, and 10,886 in year 3. The proportion of *S. pneumoniae* isolates resistant to erythromycin was similar across years 1, 2, and 3 of the PROTEKT US study (29.4% overall). The prevalence of *mef*(A) in macrolide-resistant isolates decreased from 68.8% in year 1 to 67.3% in year 2 and to 63.9% in year 3, while the prevalence of *erm*(B) alone appeared stable (16.9% in year 1, 16.5% in year 2, 16.5% in year 3). By contrast, an increase was seen in the prevalence of macrolide-resistant strains carrying both *erm*(B) and *mef*(A) genes; by year 3, 16.4% of isolates were of this genotype (Table 1). When considered as a proportion of all *S. pneumoniae* isolates collected in year 3, a total of 520 (4.8%) of 10,886 were positive for both *erm*(B) and *mef*(A).

**Table 1:** Geographic distribution by year of *Streptococcus pneumoniae* collected from years 1 to 3 of the PROTEKT US study with both *erm*(B)- and *mef*(A)-encoded macrolide resistance among genotyped erythromycin-resistant isolates

US region*	No. *erm*(B)+*mef*(A)–positive/no. erythromycin-resistant (%)			
	Year 1	Year 2	Year 3	Years 1–3 combined
North-Central	121/667 (18.1)	117/626 (18.7)	176/735 (23.9)	414/2,028 (20.4)
Northeast	88/985 (8.9)	96/771 (12.5)	140/900 (15.6)	324/2,656 (12.2)
Northwest	8/98 (8.2)	18/119 (15.1)	26/125 (20.8)	52/342 (15.2)
South-Central	23/561 (4.1)	36/463 (7.8)	80/667 (12.0)	139/1,691 (8.2)
Southeast	29/427 (6.8)	31/481 (6.4)	49/475 (10.3)	109/1,383 (7.9)
Southwest	35/395 (8.9)	37/333 (11.1)	49/275 (17.8)	121/1,003 (12.1)
Total	304/3,133 (9.7)	335/2,793 (12.0)	520/3,177 (16.4)	1,159/9,103 (12.7)

*North-Central = Illinois, Iowa, Kansas, Minnesota, Missouri, Nebraska, North Dakota, South Dakota, Wisconsin; Northeast = Connecticut, Delaware, Indiana, Maryland, Massachusetts, Michigan, New Jersey, New York, Ohio, Pennsylvania, Rhode Island, Vermont, Washington DC; Northwest = Alaska, Idaho, Montana, Oregon, Washington, Wyoming; South-Central = Alabama, Arkansas, Louisiana, Oklahoma, Tennessee, Texas; Southeast = Florida, Georgia, Kentucky, North Carolina, Puerto Rico, South Carolina, Virginia, West Virginia; Southwest = Arizona, California, Colorado, Nevada, New Mexico, Utah.

Geographic differences were observed in the prevalence of *erm*(B)+*mef*(A)–encoded resistance across the United States, from 10.3% in the Southeast to 23.9% in the North-Central region (year 3). The prevalence of this genotype increased in all regions between years 1 and 3 (Table 1).

The largest increases in *erm*(B)+*mef*(A)-encoded resistance during the 3-year study period occurred in isolates collected from pediatric patients (Table 2). By year 3, isolates exhibiting this genotype made up 254 (22.7%) of 1,119 isolates obtained from pediatric patients (≤14 years of age) compared with 98 (12.3%) of 794 isolates collected from patients >64 years of age. Patients in the 0- to 2-year age group had the highest prevalence (23.9%) of dual *erm*(B)+*mef*(A) resistance (Table 2).

**Table 2:** Proportion of erythromycin-resistant *Streptococcus pneumoniae* isolates collected from years 1 to 3 of the PROTEKT US study with the dual *mef*(A)+*erm*(B) genotype according to patient age

Patient age (y)	No. *erm*(B)+*mef*(A)–positive/no. erythromycin-resistant (%)		
	Year 1	Year 2	Year 3
0–2	88/825 (10.7)	118/640 (18.4)	170/710 (23.9)
3–14	52/388 (13.4)	47/365 (12.9)	84/409 (20.5)
15–64	98/1,106 (8.9)	95/972 (9.8)	151/1,173 (12.9)
>64	58/727 (8.0)	70/755 (9.3)	98/794 (12.3)
Not specified	8/87 (9.2)	5/61 (8.2)	17/91 (18.7)
Total	304/3,133 (9.7)	335/2,793 (12.0)	520/3,177 (16.4)

Across the 3-year study period, the dual *erm*(B)+*mef*(A) genotype was found most frequently in isolates collected from the ear or middle-ear fluid (Table 3). In year 3, the prevalence of this form of macrolide resistance was >30% in isolates collected from either of these sources. By contrast, isolates cultured from blood samples had the lowest proportion of dual *erm*(B)+*mef*(A)-encoded resistance (92 [4.6%] of 2,014 isolates in the 3 years).

**Table 3:** Proportion of erythromycin-resistant *Streptococcus pneumoniae* isolates collected from years 1 to 3 of the PROTEKT US study that exhibit the dual *mef*(A)+*erm*(B) genotype according to source of isolate*

Source of isolate	No. *erm*(B)+*mef*(A)–positive/no. erythromycin-resistant (%)		
	Year 1	Year 2	Year 3
BAL	35/346 (10.1)	47/349 (13.5)	74/426 (17.4)
Blood	24/805 (3.0)	31/568 (5.5)	37/641 (5.8)
CSF	–	1/2 (50.0)	–
Ear	67/420 (16.0)	48/246 (19.5)	107/354 (30.1)
Eye	3/16 (18.8)	11/120 (9.2)	2/7 (28.6)
MEF	2/34 (5.9)	6/29 (20.7)	14/40 (35.0)
NAP	34/246 (13.8)	43/242 (17.8)	51/235 (21.7)
Sinus	18/162 (11.1)	15/159 (9.4)	36/180 (20.0)
Sputum	117/1,056 (11.1)	129/1,050 (12.3)	199/1,294 (15.4)
Throat	1/15 (6.7)	1/8 (12.5)	–
Not specified	3/33 (9.1)	3/20 (15.0)	–
Total	304/3,133 (9.7)	335/2,793 (12.0)	520/3,177 (16.4)

*BAL, bronchoalveolar lavage; CSF, cerebrospinal fluid; MEF, middle-ear fluid; NAP, nasopharyngeal swab or aspirate; –, no isolates collected.

### Antimicrobial Resistance in Dual *erm*(B)+*mef*(A) Isolates

In addition to exhibiting almost universal resistance to the macrolides tested (azithromycin, clarithromycin, erythromycin), isolates carrying both *erm*(B) and *mef*(A) were highly resistant (>90%) to penicillin, cefuroxime, tetracycline, and co-trimoxazole (Table 4). Resistance to amoxicillin-clavulanate (and hence amoxicillin) was also common in these isolates (Table 4), and the longitudinal data showed that the rate of resistance to this antibacterial drug increased from 29.9% to 43.9% from year 1 to year 3. Almost all (1,150 [99.2%] of 1,159) of the *erm*(B)+*mef*(A) isolates were multidrug-resistant (i.e., resistant to ≥2 classes of antibacterial drugs).

**Table 4:** Susceptibility to various antibacterial drugs among *Streptococcus pneumoniae* isolates collected from years 1–3 of the PROTEKT US study that had both *erm*(B) and *mef*(A) macrolide resistance genes (n = 1,159)

Drug	% susceptibility*		
	Susceptible	Intermediate	Resistant
Amoxicillin–clavulanate†	40.6	22.0	37.4
Azithromycin	0	0.1	99.9
Cefuroxime	5.7	1.7	92.6
Clarithromycin	0	0	100
Co-trimoxazole	3.4	1.2	95.4
Erythromycin	0	0	100
Levofloxacin	98.6	0	1.4
Linezolid	99.8	0	0.2
Penicillin	1.5	6.7	91.8
Telithromycin	99.0	0.9	0.1
Tetracycline	2.7	0.7	96.6

*Susceptibility was defined according to Clinical and Laboratory Standards Institute interpretive criteria (25). †Amoxicillin alone was not tested; however, susceptibility can be extrapolated from the amoxicillin-clavulanate results.

A total of 16 (1.4%) of the 1,159 dual *erm*(B)+*mef*(A) isolates collected were resistant to levofloxacin; MIC values for these were as follows: 8 μg/mL (2 isolates), 16 μg/mL (9 isolates), 32 μg/mL (4 isolates), and 128 μg/mL (1 isolate). One dual *erm*(B)+*mef*(A) isolate (<0.1% of the total) was resistant to telithromycin (MIC 4 μg/mL).

### Molecular Epidemiology

The results of MLST determination on 518 *S. pneumoniae* isolates (366 from PROTEKT global [including 35 from the United States] and 152 from PROTEKT US) with dual *erm*(B)+*mef*(A)–encoded resistance showed 82 ST variants (Figure). Of these, 21 were in the MLST database, and 61 were submitted to the database and assigned a new ST (STs 1407–1467). All of the unique STs were serotyped and, together with the 20 *S. pneumoniae* clones listed by the Pneumococcal Molecular Epidemiology Network (29), were analyzed for clonal relatedness by using UPGMA and BURST (Figure). Both the serotype distribution and range of MLSTs in these isolates were limited (Table 5), with 3 clonal complexes predominating. A phylogenetic analysis of these variations showed that 45 of the 82 STs were closely related, either serotype 19F or 19A (Table 5). These strains were of ancestral ST 271 and hence were designated clonal complex (CC) 271, which is equivalent to CC I (15) and CC 236 (22). Overall, 305 (83.3%) of 366 global isolates had MLST profiles consistent with this clone (Table 6).

**Figure:**
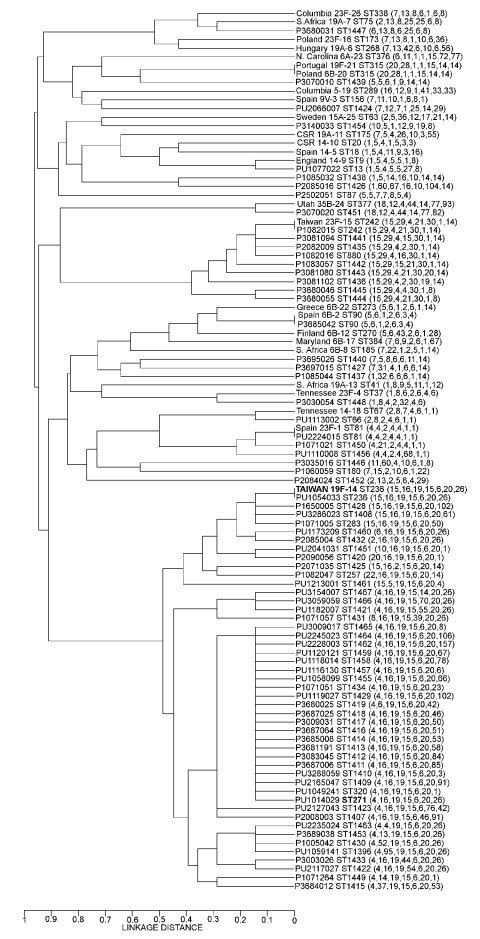
Phylogenetic relationships of the 82 different sequence type variations found in 518 *Streptococcus pneumoniae* isolates with combined *erm*(B)- and *mef*(A)-mediated macrolide resistance collected during the PROTEKT global study (1999–2003, n = 366) and the PROTEKT US study (2000–2003, n = 152) compared with the 20 PMEN (Pneumococcal Molecular Epidemiology Network [29]) clones.

**Table 5:** Distributions of sequence types and serotypes of 518 dual *erm*(B)+*mef*(A) erythromycin-resistant *Streptococcus pneumoniae* isolates collected during the PROTEKT global study (1999–2003, n = 366) and the PROTEKT US study (2000–2003, n = 152)*

Clonal complex (n)	PMEN clone designation	Sequence types (n)	Serotypes (n)
CC 271 (446)	Taiwan^19F^-14	236 (48)†, 257 (1), 271 (218), 283 (4), 320 (93), 1396 (6), 1407 (1), 1408 (2), 1409 (4), 1410 (3), 1411 (1), 1412 (3), 1413 (1), 1414 (1), 1415 (1), 1416 (2), 1417 (1),1418 (11), 1419 (4), 1420 (1), 1421 (3), 1422 (1), 1423 (1), 1425 (1), 1428 (3), 1429 (2), 1430 (1), 1431 (1), 1432 (3), 1433 (2), 1434 (1), 1449 (1), 1451 (5), 1453 (1), 1455 (2), 1457 (1), 1458 (1), 1459 (1), 1460 (1), 1461 (2), 1462 (1), 1463 (1), 1464 (2), 1465 (1), 1466 (1)	14 (3), 19A (66), 19F (376), NT (1)
CC 242 (25)	Taiwan^23F^-15	242 (9), 880 (1), 1435 (2), 1436 (1), 1441 (1), 1442 (1), 1443 (1), 1444 (7), 1445 (2)	23F (25)
CC 81 (12)	Spain^23F^-1	81 (10), 1450 (1), 1456 (1)	14 (1), 19F (3), 23F (5), 6A (1), NT (2)
Singletons		13 (4), 66 (1), 87 (2), 90 (2), 451 (2), 1424 (3), 1426 (8), 1427 (1), 1437 (2), 1438 (1), 1439 (1), 1440 (1), 1446 (1), 1447 (1), 1448 (1), 1452 (1), 1454 (1), 1467 (2)	14 (4), 34(1), 16F (1), 19A (9), 19F (6), 23F (2), 35B (2), 6A (1), 6B (7), 7F (1), 9N (1)

*PMEN, Pneumococcal Molecular Epidemiology Network; NT, nontypeable. †Sequence type 236 was previously described as CC 236 by Ko and Song (22) but is described as CC 271 in this study based on BURST analysis.

**Table 6:** Lineage by country of 366 dual *erm*(B)+*mef*(A) erythromycin-resistant *Streptococcus pneumoniae* isolates collected during the PROTEKT global study (1999–2003)

Country	No. isolates	Clonal complex			
		CC 271	CC 242	CC 81	None*
Australia	6	6	–	–	–
Brazil	2	2	–	–	–
Canada	4	4	–	–	–
China	13	12	–	–	1
France	3	1	–	–	2
Germany	1	1	–	–	–
Hong Kong	2	2	–	–	–
Hungary	2	2	–	–	–
Italy	2	2	–	–	–
Japan	44	12	16	1	15†
Mexico	6	4	–	–	2
South Africa	129	116	9	2	2
South Korea	111	102	–	6	3
Taiwan	5	3	–	–	2
United Kingdom	1	1	–	–	–
United States	35	35	–	–	–
Total, n (%)	366 (100)	305 (83.3)	25 (6.8)	9 (2.5)	27 (7.4)

*No clonal lineage found except for 2 isolates (ST 1467) and 1 isolate (ST 1452), which were clonally related (CC 1467). †8 isolates were clonally related (ST 1426).

Of the 35 isolates collected in the United States from the PROTEKT global study, all exhibited MLST profiles and serotypes characteristic of CC 271 (Table 6). Moreover, analysis of a geographically and chronologically varied sample of 152 *S. pneumoniae* isolates with dual *erm*(B)+*mef*(A)–encoded resistance collected from the PROTEKT US study suggested that >90% of dual-resistant isolates in the United States belong to CC 271.

## Discussion

Pneumococcal macrolide resistance in the United States is predominantly mediated by the *mef*(A) gene, which encodes for lower-level, efflux-mediated resistance (14). However, the latest surveillance data from PROTEKT US presented in this article show that the prevalence of this form of resistance is decreasing. This trend coincides with the emergence of multidrug-resistant clones of *S. pneumoniae* that express both *erm*(B) and *mef*(A). These strains increased in prevalence from 9.7% of macrolide-resistant isolates in 2000–2001 to 16.4% in 2002–2003. Moreover, geographic data indicate that dual *erm*(B)+*mef*(A) isolates are currently even more prevalent in some regions of the United States (accounting for >20% of macrolide-resistant strains). By 2002–2003, *S. pneumoniae* strains with this dual mechanism of resistance made up almost 5% of all isolates collected.

The major clinical implication of the present report is the increased potential for treatment failure with most antibacterial drugs currently recommended to empirically treat community-acquired respiratory tract infections (30,31). Ear isolates are more prone to represent treatment failure, and blood isolates represent primary infection; thus, the dramatic increase in CC 271 in ear isolates compared to blood isolates (Table 2) is noteworthy. Almost all dual *erm*(B)+*mef*(A) isolates were highly resistant to multiple antibacterial drugs, including penicillin, macrolides, tetracycline, and co-trimoxazole. This high-level macrolide resistance is presumably mediated by the *erm*(B) gene. Furthermore, resistance to amoxicillin-clavulanate (and hence amoxicillin) increased in these isolates from 29.9% to 43.9% during the 3-year surveillance period, which raises concerns about the potential selection of resistant isolates through widespread use of this agent for community-acquired infections, particularly acute otitis media.

The prevalence of resistance to fluoroquinolones, such as levofloxacin, was low (1.4% overall) in the dual *erm*(B)+*mef*(A) isolates; however, when present, this resistance was often high (MIC 8–128 μg/mL). Telithromycin resistance was rare (<0.1%) in *S. pneumoniae* isolates with dual *erm*(B)+*mef*(A)–encoded macrolide resistance.

Previous studies have indicated that a small number of clonal groups account for most penicillin-, macrolide-, and multidrug-resistant *S. pneumoniae* in the United States (18,32). The MLST analysis conducted in the present study shows that the dual *erm*(B)+*mef*(A) macrolide-resistant *S. pneumoniae* isolates collected in the United States from 1999 to 2003 are associated with 3 major global clones, in addition to a wide variety of other MLST variations. Most of these isolates belong to 1 major clonal group; the genotypic profile and serotype distribution of this predominant group show that it is highly related to an international *erm*(B)+*mef*(A) clonal strain, Taiwan^19F^-14, first found in the Far East (22). The designation of clonal groups is determined by BURST analysis, which assigns ancestral lineage by the most common ST. For this reason, the pneumococcal clone designated CC 271 in the present study was named CC 236 in the study by Ko and Song (22). To avoid confusion, a common CC nomenclature (such as the original designation of the clone, CC 1 [15]) may be more useful.

The pneumococcal clone discussed in this paper was previously identified in the first year (1999–2000) of the PROTEKT global study (15). The most recent data from this survey, which covered the period 1999–2003, confirm that this clone now has a worldwide distribution, with particularly high incidences in South Africa and South Korea, as reported in previous studies (15,19,21). Strains carrying both genes have also been recorded recently in New Zealand (33), Canada (34), Italy (35), and Scotland (36). Together with the regional genotyping data, the epidemiologic analyses we describe show that this multidrug-resistant CC 271 is now widespread and increasing in prevalence across the United States.

The widespread emergence of the *erm*(B)+*mef*(A) genotype into varying lineages at the apparent expense of strains expressing only 1 resistance determinant suggests that *S. pneumoniae* carrying this form of resistance has an evolutionary advantage. Since dual resistant isolates have drug MICs similar to those observed in strains harboring *erm*(B) alone, such an advantage cannot be explained on the basis of increased macrolide resistance alone. This clone has previously been shown to contain 2 mobile genetic elements, Tn*1545* and "mega" (15). While the *erm*(B) gene is most often present on Tn*1545*, "mega" is known to contain the *mef*(E) variant of *mef*(A), and this variant has been shown to be present in CC 271 (15). Hence, acquisition of 2 mobile genetic elements and associated resistance genes is a possible explanation for the successful emergence of this clone over isolates with only *erm*(B) or *mef*(A); it is not solely the acquisition of the efflux or methylase gene but the associated resistance genes on the genetic elements that lead to a multidrug-resistant clone in which prevalence is driven by greater environmental pressures.

Of particular concern is the finding that dual *erm*(B)+*mef*(A)–encoded resistance was most prevalent in isolates collected from pediatric patients. By year 3 of the study, 8.7% of all *S. pneumoniae* isolates collected from children ≤14 years of age and 10.7% of those collected from children ≤2 years of age exhibited this form of macrolide resistance. The introduction of the 7-valent pneumococcal vaccine (PCV7) in 2000 was aimed primarily at reducing the incidence of disease in this vulnerable group. While recent evidence suggests that this reduction has occurred (37,38), the vaccine does not provide coverage against all *S. pneumoniae* serotypes. As discussed above, most dual *erm*(B)+*mef*(A) isolates characterized in this study are of serotype 19A (the prevalence of which increased from years 1 to 3) or 19F. Although serotype 19F is represented in the PCV7 vaccine, it affords low levels of protection against upper respiratory infections such as otitis media (39) and has been shown recently to be the least immunogenic of the vaccine serotypes (40). Moreover, little evidence shows that 19F provides cross-protection against serotype 19A. The trends reported in this article indicate that the introduction of routine immunization has not prevented the spread of this nonvaccine serotype multidrug-resistant clone in the pediatric population and may have contributed to the selection of serotype 19A strains.

In summary, although pneumococcal macrolide resistance rates appear to have stabilized in the United States, prevalence of clonal isolates with the combined *erm*(B)+*mef*(A) genotype is increasing. These strains show high-level macrolide and multidrug resistance, and their spread across the United States represents a serious public health concern. These findings also highlight the critical need for continued monitoring of pneumococcal resistance patterns over time, in particular, the spread of these multidrug-resistant clones, and for physicians to be aware of local or regional resistance patterns when selecting empiric antibacterial treatment for community-acquired respiratory tract infections.
